# Complete Genome Sequence of Bacillus cereus Strain PL1, Isolated from Soil in Japan

**DOI:** 10.1128/MRA.00195-20

**Published:** 2020-03-19

**Authors:** Kentaro Miyazaki, Erina Hase, Tomomasa Maruya

**Affiliations:** a Department of Life Science and Biotechnology, Bioproduction Research Institute, National Institute of Advanced Industrial Science and Technology (AIST), Tsukuba, Ibaraki, Japan; b Department of Computational Biology and Medical Sciences, Graduate School of Frontier Sciences, The University of Tokyo, Kashiwa, Chiba, Japan; c Department of Engineering Science, Graduate School of Informatics and Engineering, The University of Electro-Communications, Chofu, Tokyo, Japan; University of Maryland School of Medicine

## Abstract

We isolated the soil bacterium strain PL1 and herein report its complete genome sequence. The strain presented 97% average nucleotide identity (ANI) to Bacillus cereus and 91% ANIs to other members of the B. cereus group, indicating that it is affiliated with B. cereus.

## ANNOUNCEMENT

The Bacillus cereus group includes several *Bacillus* species with close phylogenetic relationships. Among these, the species that have been studied the most are B. anthracis, B. cereus, B. thuringiensis, B. mycoides, B. pseudomycoides, B. weihenstephanensis, B. cytotoxicus, and B. toyonensis (1–3). Because some of these species are known to be potentially pathogenic (3, 4), numerous efforts have been made to clinically diagnose them. One routine method for bacterial diagnostics involves 16S rRNA gene sequencing. However, because the sequences of B. cereus group species are markedly similar, often presenting >99% identity rates, this approach is not effective for species identification among these group members. With the advent of next-generation sequencing technologies, whole-genome sequencing is becoming the prime choice when complementing the drawbacks (i.e., misidentification) of traditional 16S rRNA gene-based classification (2, 5).

To screen for soil bacteria, we collected soil samples from the city of Tsukuba, Japan. The soil sample was spread over LB agar (1% [wt/vol] tryptone, 0.5% [wt/vol] yeast extract, 0.5% [wt/vol] NaCl, and 1.5% [wt/vol] agar) plates and incubated at 37°C overnight. Several single colonies were isolated. DNA sequencing of the nearly full-length 16S rRNA genes revealed that many of them were affiliated with one of the following genera: *Bacillus*, *Streptomyces*, or *Klebsiella*. The 16S rRNA sequence from PL1 showed ∼99.9% similarity to that of B. wiedmannii (GenBank accession number NR_152692.1), B. cereus (NR_115714.1), and B. proteolyticus (NR_157735.1). To more accurately understand the taxonomic position of the strain, we subjected it to whole-genome analysis. We used a hybrid approach involving a combination of long-read sequencing with a GridION device (Oxford Nanopore Technologies [ONT]) and short-read sequencing with a MiSeq instrument (Illumina). Software analyses were conducted using default parameter settings throughout this study.

The PL1 strain was grown in LB broth at 37°C for 18 h. Genomic DNA was extracted following a previously described procedure (6). For long-read sequencing, genomic DNA (1 μg) was treated with Short Read Eliminator XS (Circulomics). The resulting DNA was used to construct a library using a ligation sequencing kit (SQK-LSK109; ONT), and the library was analyzed on a FLO-MIN106 R9.41 flow cell (ONT) for 9 h. Base calling was conducted using Guppy v.3.3.2 (ONT) to generate 172,649 reads (935 Mb) with an average length of 5,418.1 bases. The raw reads were filtered (Q ≥ 10, read length ≥ 1,000 bases) using NanoFilt v.2.3.0 (7). The longest read contained 104,681 bases.

For short-read sequencing, the Nextera DNA Flex library prep kit (Illumina) was used to generate paired-end libraries with approximately 350-bp inserts. Sequencing was performed using a MiSeq reagent kit v.2 (300 cycles) with reads that were 156 bp long. Adapter sequences and low-quality data were trimmed (Q ≥ 30, read length ≥ 10 bases) using fastp v.0.20.0 (8), yielding 436 Mb of data containing 1.46 million paired-end reads with an average length of 149.8 bp.

The long-read and short-read data were assembled *de novo* using Unicycler v.0.4.8 (9), followed by polishing with Pilon v.1.23 (10). This yielded a single circular chromosome (5,309,441 bp) and six putative plasmids (pBwiPL1-1 to pBwiPL1-6). The obtained sequence data were submitted to a Web-based annotation pipeline, DFAST v.1.2.4 (11), for automated annotation. The chromosome contained 5,308 protein-coding, 106 tRNA, and 42 rRNA genes. Other characteristics are summarized in Table 1. Average nucleotide identity (ANI) was analyzed using the JSpeciesWS online service (12), which revealed that the PL1 genome sequence had 97% ANI to B. cereus (GenBank accession number NC_004722), ∼91% to other B. cereus group species (B. mycoides, B. toyonensis, B. wiedmannii, and B. anthracis), and ∼82% to B. pseudomycoides and *B. cytotoxicus*, conclusively indicating the taxonomic affiliation of PL1 with B. cereus.

**TABLE 1:** Genome statistics and features of Bacillus cereus strain PL1

Chromosome or plasmid	Length (bp)	GC content (%)	No. of CDSs^*a*^	No. of rRNAs	No. of tRNAs	Avg read depth (×)	Accession no.
Chromosome	5,309,441	35.3	5,308	42	106	70.4	AP022643
Plasmid, pBwiPL1-1	363,764	32.5	311	0	0	94.6	AP022644
Plasmid, pBwiPL1-2	72,105	34.4	102	0	0	67.7	AP022645
Plasmid, pBwiPL1-3	60,534	43.7	77	0	0	111.0	AP022646
Plasmid, pBwiPL1-4	14,157	38.9	21	0	0	605.7	AP022647
Plasmid, pBwiPL1-5	13,909	41.0	12	0	0	296.2	AP022648
Plasmid, pBwiPL1-6	8,882	32.1	7	0	0	323.8	AP022649

a CDSs, coding DNA sequences.

### Data availability.

The complete genome sequence of B. cereus PL1 is available from DDBJ/EMBL/GenBank under the accession numbers AP022643 (chromosome) and AP022644 to AP022649 (plasmids). Raw sequencing data have been deposited in the DDBJ SRA database under the accession number DRA009574 (BioProject PRJDB9286, BioSample SAMD00204526).
